# Theoretical Study on Non-Linear Optics Properties of Polycyclic Aromatic Hydrocarbons and the Effect of Their Intercalation with Carbon Nanotubes

**DOI:** 10.3390/molecules28010110

**Published:** 2022-12-23

**Authors:** Imane Khelladi, Michael Springborg, Ali Rahmouni, Redouane Chadli, Majda Sekkal-Rahal

**Affiliations:** 1 Laboratoire de Chimie Théorique de Bio- et Nanosystemes, Faculty of Exact Sciences, University Djillali Liabes of Sidi Bel-Abbes, B.P. 89, Sidi Bel Abbes 22000, Algeria; 2 Modeling and Computational Methods Laboratory, University of Saida, B.P. 148, Cité En-Nasr, Route de Mascara, 2002, Saida 20000, Algeria; 3 Laboratory of Theoretical Chemistry, Department of Chemistry, Namur Institute of Structured Matter (NISM), University of Namur, Rue de Bruxelles 61, 5000 Namur, Belgium

**Keywords:** conjugated molecules, non-linear optical responses, functionalization, embedding

## Abstract

Results of a theoretical study devoted to comparing NLO (non-linear optics) responses of derivatives of tetracene, isochrysene, and pyrene are reported. The static hyperpolarizability β, the dipole moment μ, the HOMO and LUMO orbitals, and their energy gap were calculated using the CAM-B3LYP density functional combined with the cc-pVDZ basis set. The para-disubstituted NO_2_-tetracene-N(CH_3_)_2_ has the highest NLO response, which is related to a large intramolecular charge transfer. Adding vinyl groups to the para-disubstituted NO_2_-tetracene-N(CH_3_)_2_ results in an increase in the NLO responses. We further investigated the effect of the intercalation of various push–pull molecules inside an armchair single-walled carbon nanotube. The intercalation leads to increased NLO responses, something that depends critically on the position of the guest molecule and/or on functionalization of the nanotube by donor and attractor groups.

## 1. Introduction

The backbone of polycyclic aromatic hydrocarbons (PAHs) contains a sequence of at least two fused benzene rings whereby the way they are linked distinguishes different PAHs [1,2]. PAHs can have an unlimited number of contiguous rings [3,4,5]. This gives rise to a large number of isomers and enriches this family of aromatic hydrocarbons. The main approach for producing PAHs is through an incomplete combustion of organic materials (for instance, fuels and coal) [6,7,8]. PAHs are divided into two classes (light and heavy) according to the number of rings involved in their structures. Each class has its own physicochemical properties [9], which allows for a large variety of different applications including organic field effect transistors [10,11,12], organic light-emitting diodes [13], reinforcing agents in pigment lasers [14], and batteries [15].

The aim of the present study is to use theoretical methods to study the performance of three smaller PAHs, i.e., tetracene, isochrysene (or triphenylene), and pyrene, with special emphasis on their non-linear optics (NLO) responses. The π electrons of these conjugated molecules [16,17,18] facilitate an intramolecular charge transfer (ICT) between electron donor (D) and electron acceptor (A) groups when such groups are attached [19,20]. To study how the NLO responses can be influenced upon functionalization of the system is one purpose of the present work. 

A number of recent papers have focused on intramolecular charge transfer in PAHs, including studies on tetracyclic molecules and their derivatives [21,22,23,24,25]. Moreover, it has been shown that purely organic rings can be considered as being more aromatic than BN-containing systems [26] and, accordingly, to have more delocalized π electrons. 

Even carbon nanotubes (CNTs) can be considered as being a special case of extended PAHs, independent of whether they are single-walled carbon nanotubes, SWCNTs; or multi-walled carbon nanotubes, MWCNTs. Since their discovery in 1991 [27], a vast number of studies of their properties have appeared, including studies of their practical applications in, e.g., pharmacy, mechanics, and optoelectronics. They possess a high mechanical resistance, a high electrical and thermal conductivity, and chemical inertness [28,29,30]. Because of their optoelectronic properties, they have been used for light-emitting diodes [31]. In addition, functionalization of SWCNTs has been used as a way of improving their properties as shown, e.g., by Khazaei et al. [32]. The hollow structure of the carbon nanotubes, shared by the fullerenes, allows for intercalation, a prospect that has been studied by, e.g., Hirscher et al. [33] and by Chaban et al. [34,35]. 

In addition, NLO properties of such systems have been at the center of earlier studies [36,37]. However, a more systematic study of the dependence of the NLO properties on the size of the system, on functionalization, and on intercalation is lacking, although this could provide very useful information for experimentalists who aim at designing optimal systems. It is the purpose of the present work to provide results of such a study.

**Figure 1:**
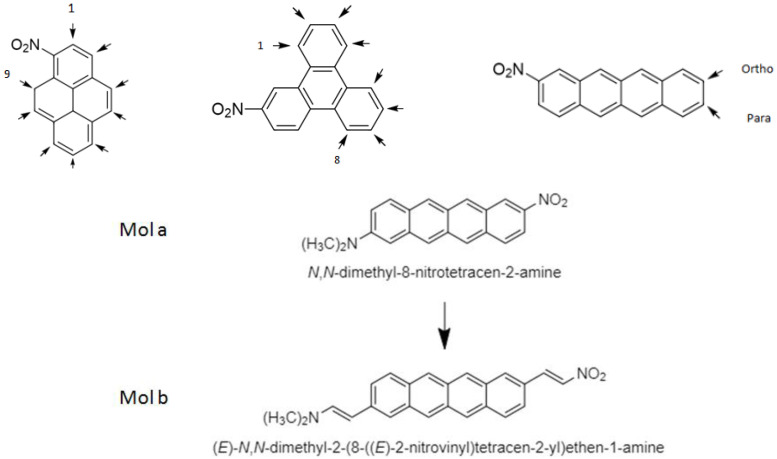
Schematic representation of the molecules of the present study. The arrows show positions at which functional groups are attached.

We also study some push–pull molecules when interacting with SWCNTs. The push–pull molecules considered in this work are shown on Figure 1. They consist of the pure polycyclic aromatic hydrocarbons tetracene, isochrysene, and pyrene and they all contain a conjugated bridge with delocalized π electrons [38,39,40]. We study the effects of substituting the PAHs at different positions through donors [41]: NH_2_, N_2_H_3_, N(CH_3_)_2_, OH, OCH_3_, and the acceptor group NO_2_ [42]. 

For the molecule giving the largest NLO response, i.e., tetracene, we subsequently studied modified versions of this containing a larger conjugated part. This was achieved by the addition of vinyl groups at the terminations of tetracene. Then, we compared the basic molecule (Mol a) and the derivative obtained after the modification (Mol b) in terms of intramolecular charge transfer, the first hyperpolarizability, and the dipole moment.

**Figure 2:**
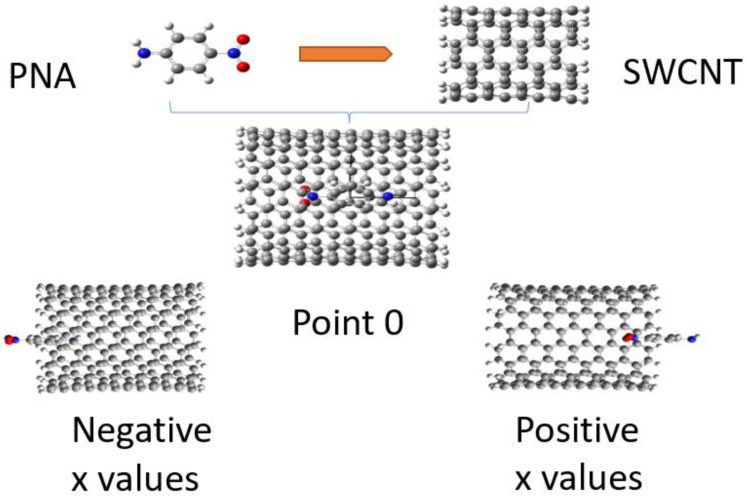
Structures showing the positions of the guest molecule (PNA) inside the host molecule (SWCNT).

**Figure 3:**
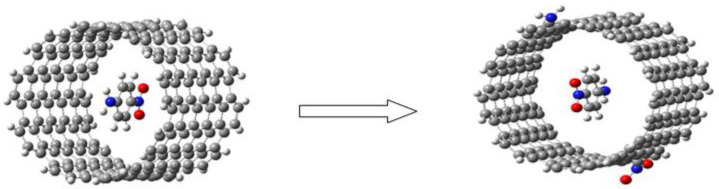
Armchair-type nanotube structure to which a donor and an electron acceptor group have been attached.

Subsequently, we considered the effects of intercalation of derivatives of a single PAH molecule inside carbon nanotubes. Initially, we constructed a (9,9) armchair nanotube with a diameter of 12.21 nm and a length of 19.69 nm as shown, e.g., in Figure 2 and Figure 3 Dangling bonds at the ends were saturated with hydrogen atoms. The initial structure of this chair-type SWCNT was optimized using the B3LYP density functional [43] combined with the 6-31g(d,p) basis set [44]. We investigated the effect of the position of the guest molecule, paranitroaniline (PNA), inside the nanotube by performing single-point calculations using the CAM-B3LYP functional together with the GD3 dispersion correction [45,46] and using the 6-31g(d,p) basis set. Various NLO parameters, including the static first hyperpolarizability, the dipole moment, and the HOMO–LUMO energy gap, were calculated for different positions of the guest molecule inside the nanotube by translating the former along the x-axis (parallel to the tube) with a step length of 2 Å relative to the initial position (denoted position 0, cf., Figure 2). 

**Figure 4:**
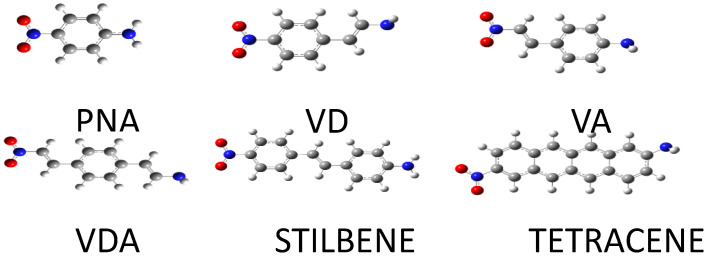
Structures of the push–pull molecules that were inserted inside the armchair-type carbon nanotube.

After that, we examined the effect of the size of the guest molecule on the intramolecular charge transfer of the system. For that purpose, we considered different push–pull molecules inserted inside the chair-like nanotube. As guest molecules, we considered PNA, VD, VA, VDA, stilbene, and tetracene, all shown in Figure 4. For each system, the static hyperpolarizabilities and the dipole moment were calculated

Finally, we modified the host system, i.e., to the armchair-type nanotube, we attached an NH_3_ donor on one side and an NO_2_ acceptor group on the other side, cf., Figure 3. At first, the structure of the isolated host was optimized using B3LYP/6-31g(d,p), after which the push–pull molecule PNA was inserted in the center of the tube and calculations were performed to check the effect of these substitutions on the hyperpolarizabilities and on the total dipole moment.

## 2. Computational Details

At first, we emphasize that our study involves several approximations. The size and number of the systems of our interest make it prohibitive to apply the most accurate computational methods for each of those. Instead, our focus is on studying the changes when modifying the systems in one way or another, so that our results should be able to describe those changes, although the absolute numbers will be less accurate. The approximations we employ include a basis set of finite size, the finite lengths of the carbon nanotubes, and the density functional itself.

All structures were visualized using the Gaussview 5.0 software [47] and all calculations were performed using Gaussian 09 [48]. Geometry optimizations of all the molecules and their derivatives were carried out using the density functional B3LYP [49,50,51] combined with the cc-pVDZ basis set [52,53,54].

Initially, we performed a benchmark study comparing static hyperpolarizabilities $\beta_{tot}$ obtained using a number of hybrid functionals such as PBE0, BMK, BHHLYP, M062X, and CAM-B3LYP in which the Hartree–Fock exchange is partly incorporated, i.e., to 25%, 42%, 50%, 54%, and 65%, respectively [55,56,57,58,59]. The results were compared to those obtained using the MP2 method (the second-order Møller−Plesset perturbation method). The latter was taken as a reference due to the absence of experimental values as MP2 results often are considered accurate [60,61,62,63]. This comparison was performed only on tetracene derivatives in order to determine the appropriate functional for this kind of system and for the first static hyperpolarizability β (cf., Equation (1)) [64,65,66]. 

In the general case, the first hyperpolarizability is a 3 × 3 × 3 tensor that, however, can be reduced to 10 numbers with the help of the Kleinman notation, *β_xxx_, β_xxy_, β_xyy_, β_yyy_, β_xxz_, β_xyz_, β_yyz_, β_xzz_, β_yzz_*, and *β_zzz_* [67].

We focused on the total hyperpolarizability:

(1) $$\beta_{tot}={\left(\beta_{x}^{2}+\beta_{y}^{2}+\beta_{z}^{2}\right)}^{\frac{1}{2}}$$

with

(2) $$\beta_{x}=\left(\beta_{xxx}+\beta_{xyy}+\beta_{xzz}\right)$$

(3) $$\beta_{y}=\left(\beta_{yyy}+\beta_{yzz}+\beta_{yxx}\right)$$

(4) $$\beta_{z}=\left(\beta_{zzz}+\beta_{zyy}+\beta_{zxx}\right)$$

According to our benchmark study, the CAM-B3LYP functional provides the best agreement with the MP2 reference results. Therefore, this functional was used in the subsequent calculations. This finding agrees with that of Rabah et al. [52].

Subsequently, we performed single-point (SP) calculations using the CAM-B3LYP functional combined with the cc-pVDZ basis set on each molecule. This functional includes a description of long-range corrections [68,69] and, accordingly, it provides a better description of properties related to an intramolecular charge transfer [70].

The dipole moment was calculated according to [71,72]

(5) $$\mu =\sqrt{\left(\mu_{x}^{2}+\mu_{y}^{2}+\mu_{z}^{2}\right)}$$

We also used the energy gap between the HOMO and the LUMO frontier orbitals:

(6) $$\Delta E_{H-L}=\varepsilon_{LUMO}-\varepsilon_{HOMO}$$

as parameters quantifying the NLO properties of our systems. 

For the geometric structure, we focused on the BLA (Bond Length Alternation) parameter, i.e., the difference between the average lengths of single and double bonds in a conjugated system [73]. A smaller value of the BLA facilitates an intramolecular charge transfer. 

## 3. Results and Discussion

### 3.1. I-NLO Responses of PAHs

#### 3.1.1. Ia. Selection of the Functional 

In this part, we identify the density functional that gives results closest to those obtained with the MP2 method. The latter is considered as reliable for NLO properties. DFT (functionals BMK, BHHLYP, CAM-B3LYP, M062X, and PBE0) as well as MP2 calculations were performed in combination with the cc-pVDZ basis set to calculate the first hyperpolarizabilities of ten tetracene derivatives. 

The results (see Table 1) show that the PBE0 functional overestimates the hyperpolarizabilities. The values related to the functionals BMK, BHHLYP, and M062X give a less pronounced difference, whereas the best agreement is obtained for the functional CAM-B3LYP. This is explained by the fact that this functional includes long-range Hartree–Fock exchange interactions. Consequently, the subsequent calculations for the pyrene and isochrysene derivatives were carried out using this functional in combination with the cc-PVDZ basis set. That this combination yields accurate results, particularly concerning trends, is in agreement with our earlier findings [52].

#### 3.1.2. Ib. Study of Intramolecular Charge Transfer

The calculated static first-order hyperpolarizabilities reported in Table 2 show that among the tetracene derivatives, the para-disubstituted NO_2_-tetracene-N(CH_3_)_2_ gives the largest value of β as well as the largest dipole moment µ, and also the lowest-energy gap, which is roughly inversely proportional to an intramolecular charge transfer. It is added that a comparison of the dipole moment or the hyperpolarizability between different molecules is hampered by the fact that these properties are extensive properties, so, in general, larger molecules have larger values for these properties. However, the differences we discuss here are larger than what can be explained through this simple fact.

We studied all isochrysene derivatives containing the NO_2_ group at one side of the chromophore and an electron donor (i.e., NH_2_. N(CH_3_)_2_, N_2_H_3_, OH, or OCH_3_) at the other side (cf., Figure 1). In Table 3, we present only the results of the NLO parameters of the derivatives in which the position of the donor N(CH_3_)_2_ was varied while that of the NO_2_ group was kept fixed. This combination results in a larger ICT compared to the other combinations.

From Table 3, we can observe that the charge transfer occurs mainly along the x-axis (the main axis of the chromophore). Indeed, the value of β_y_ is very small compared to the value of β_x_, and the value of β_z_ vanishes. For substitutions at positions I-6, the largest charge transfer is obtained as the donor and the acceptor groups are parallel to the dipole moment (x-axis).

Table 4 reports results obtained for pyrene derivatives substituted with N(CH_3_)_2_ as a donor and NO_2_ as an acceptor. The results are very similar to those reported in Table 3 and we, again, notice that the charge transfer occurs along the x-axis and that the substitution at positions 1-6 gives the largest charge transfer.

Table 5 summarizes the results for those derivatives of the three molecules of our interest that possess the highest values for the first hyperpolarizability. We notice that the hyperpolarizability of the tetracene derivative is markedly larger than those of the other two derivatives. The same holds for the dipole moment. The energy gap of the tetracene derivative is smaller, which correlates with the larger charge transfer between donor and acceptor.

As the tetracene derivatives give the highest charge transfer among the three molecules, only this system is considered in the next step. In this, the π-conjugated system is extended by adding vinyl groups at either termination of the molecule (see Figure 1), so the effect of extending the π-chain length on the ICT can be analyzed [74].

This substitution leads to an increase in the first static hyperpolarizability from 85.07 10^−30^ esu to 229.79 10^−30^ esu. In addition, the dipole moment, which depends on the ICT, increases from 9.72 to 11.31 Debye.

**Figure 5:**
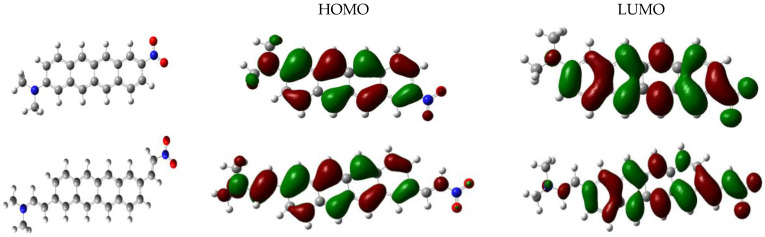
HOMO and LUMO frontier orbitals of (**top**) Mol_a and (**bottom**) Mol_b molecules.

For the energy gap, we notice only a smaller decrease from 4.53 eV for Mol_a to 4.34 eV for Mol_b. The HOMO–LUMO gap is inversely proportional to the ICT [75]. The very similar values for the gap for the two molecules can be understood from Figure 5: the frontier orbitals are largely localized to the backbone of the molecules. Equivalently, the energies of the HOMO and LUMO orbitals decrease only slightly for the substituted molecules that have a larger conjugation. 

The BLA (Bond Length Alternation) parameter is useful in quantifying NLO responses for conjugated molecules. The results reported in Table 6 show an increase in BLA upon an increase in the conjugated bridge, which correlates with the previous results. 

### 3.2. II-Intercalation inside SWCNTs

#### 3.2.1. IIa. Effect of Position 

**Figure 6:**
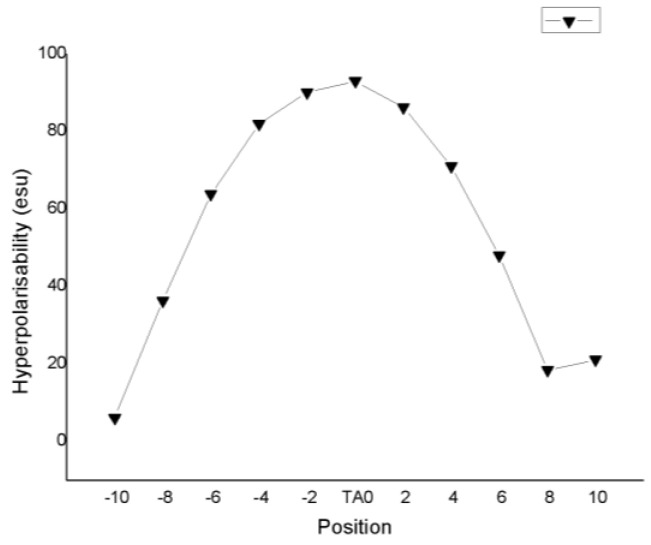
Evolution of hyperpolarizability (10^−30^ esu) of CNT-PNA as a function of the PNA position inside the CNT and as obtained with the CAM-B3LYP/6-31g(d,p) method.

The variation in the different NLO parameters as a function of the position of the paranitroaniline guest molecule inside the carbon nanotube (CNT) is reported in Table 7 and is depicted in Figure 6. According to these results, the charge transfer is largest when the guest molecule is placed in the center of the carbon nanotube, resulting in a maximum value of the static hyperpolarizability. At that position, there is a maximum guest–host interaction. The energy gap hardly varies by varying the position, a finding that is related to the fact that the two frontier orbitals HOMO and LUMO are localized mainly on the finite SWCNTs. Finally, the ICT hardly changes with the position of the guest molecule inside the CNT.

#### 3.2.2. IIb. Effect of the Nature of the Guest Molecule

In Table 8, we list the values of the first static hyperpolarizability, the dipole moment, and the energy gap for various guests inside the SWCNT. In all cases, the guest is placed at the center of the host. From these results, we observe that the VDA molecule possesses the highest hyperpolarizability despite this not being the largest molecule. The same observation holds true for the dipole moment. These high values are partly due to the longer conjugation because of the vinyl groups on either side of the benzene in the push–pull molecule, as demonstrated in the first part of this study. We also notice that the HOMO–LUMO gap remains constant for the six systems, which, again, can be explained from the localization of those two orbitals to the finite SWCNT.

#### 3.2.3. IIc. Effects due to Substitution

Finally, we considered the effects of modifying the SWCNT by adding a donor and an acceptor group to its ends (see Figure 3). This was expected to lead to an increase in the charge transfer properties of the whole system. The results are reported in Table 9. Upon substitution, β_tot_ becomes 3 times larger. Similarly, the dipole moment increases significantly, a behavior that is observed in all three spatial directions, x, y, and z. After the substitution, the value of the energy gap decreases only slightly from 1.85 to 1.82 eV.

## 4. Conclusions

The purpose of the present work was to study the effects of functionalization and/or embedding on the NLO properties of some PAHs. Therefore, our focus was not on obtaining very accurate values for specific systems, but on monitoring the changes when modifying the system of interest. 

At first, we showed that the functional CAM-B3LYP provided the most accurate description of the properties of interest when using MP2 results as a reference. Furthermore, this was most important for PAHs for which the rings are arranged linearly, as demonstrated in the case of tetracene, a case where long-ranged (exchange) interactions are most pronounced. Moreover, the addition of vinyl groups to the conjugated π bridge led to enhanced NLO responses. 

The intercalation of the PAH-derived molecules inside carbon nanotubes also led to increased NLO responses. Finally, the functionalization of the CNT through donor and acceptor groups to the CNT made it possible to increase the intramolecular charge transfer, leading to increased values of the hyperpolarizability and of the dipole moment but, in parallel, an only slightly reduced value of the energy gap.

## Figures and Tables

**Table 1:** First hyperpolarizability of tetracene derivatives (in 10^−30^ esu) using different functionals with the cc-pVDZ basis set (O: Ortho; P: Para positions of substitutions).

	NH2 -O	NH2 -P	N(CH3)2 -O	N(CH3)2 -P	OH -O	OH -P	OCH3 -O	OCH3 -P	N2H3 -O	N2H3 -P
BMK	46.71	34.64	65.77	11.01	35.68	49.82	40.66	58.78	54.94	84.75
BHHLYP	41.89	32.22	56.49	92.62	32.58	44.90	36.40	51.62	48.09	73.02
CAM-B3LYP	39.95	28.55	54.45	85.07	30.62	41.38	34.52	47.83	46.31	67.71
PBE0	84.29	67.14	120.27	235.47	67.49	104.58	78.81	129.36	100.44	179.01
M06-2X	40.68	28.25	57.43	25.78	30.28	41.22	34.49	48.14	47.84	70.89
MP2	45.65	29.78	63.76	91.29	34.06	42.51	38.57	49.16	52.83	71.22

**Table 2:** Calculated hyperpolarizability (10^−30^ esu), dipole moment (Debye), and energy gap (eV) of tetracene derivatives with substitutions at the ortho and para positions using CAM-B3LYP/cc-PVDZ. The system with the largest value for β_tot_ is highlighted.

	NH2-O	NH2-P	N(CH3)2-O	N(CH3)2-P	OH-O	OH-P	OCH3-O	OCH3-P	N2H3-O	N2H3-P
***β_x_***	39.89	−28.52	54.42	−85.01	30.60	41.29	34.52	47.78	46.28	−67.63
***β_y_***	2.06	−1.41	1.63	−3.02	0.93	−2.61	0.38	−2.30	1.13	−3.12
***β_z_***	0.68	0.01	−0.62	−0.49	0.00	0.00	0.00	0.00	−1.05	−1.12
***β_tot_***	39.95	28.55	54.45	85.07	30.61	41.38	34.52	47.83	46.31	67.71
***µ_tot_***	8.34	6.53	9.21	9.72	7.68	7.53	7.02	7.62	9.19	10.07
**gap**	4.61	4.76	4.50	4.50	4.66	4.68	4.60	4.62	4.58	4.61

**Table 3:** Calculated hyperpolarizability (10^−30^ esu), dipole moment (Debye), and energy gap (eV) of the molecule N(CH_3_)_2_-isochrysene-NO_2_ by varying the position of N(CH_3_)_2_ from 1 to 8 (denoted mol 1 to mol 8) relative to the position of NO_2,_ as obtained using CAM-B3LYP/cc-PVDZ. The system with the largest value for β_tot_ is highlighted.

	mol 1	mol 2	mol 3	mol 4	mol 5	mol 6	mol 7	mol 8
***β_x_***	−8.53	−4.81	−9.17	10.17	−9.91	13.21	9.65	−7.32
***β_y_***	−1.15	−7.96	−6.66	−4.72	−0.38	−3.05	−4.28	−4.05
***β_z_***	0.00	0.00	0.00	0.00	0.00	0.00	0.00	0.00
***β_tot_***	8.60	9.29	11.32	11.21	9.91	13.55	10.55	8.36
***µ_tot_***	5.10	4.81	8.14	7.23	7.85	5.63	7.27	7.10
**gap**	6.48	6.34	6.35	6.38	6.33	6.39	6.33	6.40

**Table 4:** Calculated hyperpolarizability (10^−30^ esu), dipole moment (Debye), and energy gap (eV) of the molecules N(CH3)_2_-pyrene-NO_2_ by varying the position from 3 to 9 of N(CH3)_2_ relative to that of NO_2_ (Position 1) giving mol 1-3 to mol 1-9, using CAM-B3LYP/cc-PVDZ. The system with the largest value for β_tot_ is highlighted.

	mol 1-3	mol 1-4	mol 1-5	mol 1-6	mol 1-7	mol 1-8	mol 1-9
***β_x_***	3.83	−10.94	−19.48	−22.99	19.08	19.04	−10.89
***β_y_***	9.42	−1.18	0.79	2.69	−1.44	−1.09	−1.79
***β_z_***	0.60	0.29	0.67	0.75	0.43	0.59	0.15
***β_tot_***	10.18	11.01	19.50	23.16	19.14	19.08	11.03
***µ_tot_***	4.92	5.91	6.65	6.89	7.62	6.20	5.02
**gap**	5.26	5.42	5.48	5.25	5.38	5.21	5.41

**Table 5:** Comparison between results for tetracene, isochrysene, and pyrene derivatives (first static hyperpolarizability in 10^−30^ esu, dipole moment (Debye), energy gap (eV), and energies of the HOMO and LUMO orbitals in eV) as obtained using CAM-B3LYP and cc-pVDZ.

	*β_0_*	*µ*	gap	HOMO	LUMO
**D-TETR-A**	85.07	9.72	4.53	−6.13	−1.61
**D-ISO-A**	13.55	5.63	6.39	−7.67	−1.28
**D-PYR-A**	23.16	6.89	5.25	−6.75	−1.50

**Table 6:** Comparison between the results for tetracene and divinyl-tetracene derivatives: first static hyperpolarizability in 10^−30^ esu, dipole moment (Debye), energy gap (eV), HOMO and LUMO orbital energies in eV, and BLA in Å as obtained with CAM-B3LYP and cc-pVDZ.

	*β*_0_	*µ*	HOMO	LUMO	GAP	BLA
**Mol_a**	85.07	9.72	−6.25	−1.64	4.53	0.077
**Mol_b**	229.79	11.31	−6.14	−1.72	4.34	0.086

**Table 7:** Calculated hyperpolarizability (10^−30^ esu), dipole moment (Debye), and energy gap (eV) of CNT-PNA, using the CAM-B3LYP/6-31g(d,p) method as a function of the position (in Å) of the PNA inside the SWCNT.

	−10	−8	−6	−4	−2	0	2	4	6	8	10
***β_tot_***	6.14	36.37	63.82	81.97	92.14	93.00	86.28	71.12	47.99	18.46	21.09
***µ_tot_***	5.18	3.97	3.09	2.56	2.36	2.33	2.45	2.86	3.72	5.29	7.24
**gap**	1.85	1.85	1.85	1.85	1.85	1.85	1.85	1.85	1.85	1.85	1.85

**Table 8:** Calculated hyperpolarizability (10^−30^ esu), dipole moment (Debye), and energy gap (eV) of different CNT-guest molecules, using CAM-B3LYP/6-31g(d,p).

	PNA	STIL	TETR	VA	VD	VDA
***β_tot_***	93.00	89.60	68.79	78.82	72.49	114.35
***µ_tot_***	2.32	3.46	2.75	2.15	1.97	3.57
**gap**	1.85	1.85	1.85	1.85	1.85	1.85

**Table 9:** Calculated hyperpolarizability (10^−30^ esu), dipole moment (Debye), and energy gap (eV) of SWCNT-PNA with (denoted TA1) and without (denoted TA0) substitution on the SWCNT, as obtained from the CAM-B3LYP/6-31g(d,p) calculations.

	TA0	TA1
***β_x_***	−7.92	262.94
***β_y_***	0.63	−101.40
***β_z_***	−92.66	−19.93
***β_tot_***	93.00	282.52
***µ_x_***	−2.31	6.78
***µ_y_***	0.09	5.82
***µ_z_***	0.23	0.42
***µ_tot_***	2.32	8.94
**Gap**	1.85	1.82

## Data Availability

The data can be obtained from IK upon request.

## References

[1] Young D., Douglas K.M., Eiceman G.A., Lake D.A., Johnston M.V. (2002). Laser desorption–ionization of polycyclic aromatic hydrocarbons from glass surfaces with ion mobility spectrometry analysis. Anal. Chim. Acta.

[2] Ravindra K., Sokhi R., Grieken R.V. (2008). Atmospheric polycyclic aromatic hydrocarbons: Source attribution, emission factors and regulation. Atmos. Environ..

[3] Alajtal A.I., Edwards H.G.M., Elbagerma M.A., Scowen I.J. (2010). The effect of laser wavelength on the Raman Spectra of phenanthrene, chrysene, and tetracene: Implications for extra-terrestrial detection of polyaromatic hydrocarbons. Spectrochim. Acta Part A.

[4] Feng X., Pisula W., Müllen K. (2009). Large polycyclic aromatic hydrocarbons: Synthesis and discotic organization. Pure Appl. Chem..

[5] Arey J., Atkinson R., Douben P.E.T. (2003). Photochemical Reactions of PAHs in the Atmosphere. PAHs: An Ecotoxicological Perspective.

[6] Lima A.L.C., Farrington J.W., Reddy C.M. (2005). Combustion-Derived Polycyclic Aromatic Hydrocarbons in the Environment—A Review. Environ. Forensics.

[7] Du J., Jing C. (2011). Preparation of Thiol Modified Fe_3_O_4_@Ag Magnetic SERS Probe for PAHs Detection and Identification. J. Phys. Chem. C.

[8] Broniatowski M., Binczycka M., Wójcik A., Flasiński M., Wydro P. (2017). Polycyclic aromatic hydrocarbons in model bacterial membranes–Langmuir monolayer studies. BBA-Biomembranes.

[9] Abdel-Shafy H.I., Mansour M.S.M. (2016). A review on polycyclic aromatic hydrocarbons: Source, environmental impact, effect on human health and remediation. Egypt. J. Pet..

[10] Bayn A., Feng X., Müllen K., Haick H. (2013). Field Effect Transistors Based on Polycyclic Aromatic Hydrocarbons for the Detection and Classification of Volatile Organic Compounds. ACS Appl. Mater. Interfaces.

[11] Chien C., Lin C., Watanabe M., Lin Y., Chao T., Chiang T., Huang X., Wen Y., Tu C., Sun C. (2012). Tetracene-based field-effect transistors using solution processes. J. Mater. Chem..

[12] Wünsche J., Tarabella G., Bertolazzi S., Bocoum M., Coppedè N., Barba L., Arrighetti G., Lutterotti L., Iannotta S., Cicoira F. (2013). The correlation between gate dielectric, film growth, and charge transport in organic thin film transistors: The case of vacuum-sublimed tetracene thin films. J. Mater. Chem. C.

[13] Hashimoto S., Ikuta T., Shiren K., Nakatsuka S., Ni J., Nakamura M., Hatakeyama T. (2014). Triplet-Energy Control of Polycyclic Aromatic Hydrocarbons by BN Replacement: Development of Ambipolar Host Materials for Phosphorescent Organic Light-Emitting Diodes. Chem. Mater..

[14] Abd-El-Aziza A.S., Dalgakiran S., Kucukkaya I., Wagner B.D. (2013). Synthesis, electrochemistry and fluorescence behavior of thiophene derivatives decorated with coumarin, pyrene and naphthalene moieties. Electrochim. Acta.

[15] Wang G., Huang B., Liu D., Zheng D., Harris J., Xue J., Qu D. (2018). Exploring polycyclic aromatic hydrocarbons as an anolyte for nonaqueous redox flow batteries. J. Mater. Chem. A.

[16] Anne F.B., Purpan F.D., Jacquemin D. (2013). Charge-Transfer in Quasilinear Push-Pull Polyene Chains. Chem. Phys. Lett..

[17] Derrar S.N., Sekkal-Rahal M., Derreumaux P., Springborg M. (2014). Theoretical study of the NLO responses of some natural and unnatural amino acids used as probe molecules. J. Mol. Model..

[18] Derrar S.N., Sekkal-Rahal M., Guemra K., Derreumaux P. (2012). Theoretical study on a series of push–pull molecules grafted on methacrylate copolymers serving for nonlinear optics. Int. J. Quantum Chem..

[19] Wang S., Kim S. (2009). Photophysical and electrochemical properties of D–π–A type solvatofluorchromic isophorone dye for pH molecular switch. Curr. Appl. Phys..

[20] Kleinpeter E., Stamboliyska B.A. (2009). Hyperpolarizability of donor–acceptor azines subject to push–pull character and steric hindrance. Tetrahedron.

[21] Tang X. (2017). Theoretical study on electron structure and charge transportproperties of tetraazapentacene derivatives. J. Mol. Graph. Model..

[22] Malloci G., Mulas G., Cappellini G., Fiorentini V., Porceddu I. (2005). Theoretical electron affinities of PAHs and electronic absorption spectra of their mono-anions. Astron. Astrophys..

[23] Cardia R., Malloci G., Bosin A., Serra G., Cappellini G. (2016). Computational investigation of the effects of perfluorination on the charge-transport properties of polyaromatic hydrocarbons. Chem. Phys..

[24] Chai S., Huang J. (2015). Impact of the halogenated substituent on electronic and charge transport properties of organic semiconductors: A theoretical study. Comput. Theor. Chem..

[25] Sancho-García J.C., Pérez-Jiménez A.J. (2010). A theoretical study of p-stacking tetracene derivatives as promising organic molecular semiconductors. Chem. Phys. Lett..

[26] Ghosh D., Periyasamy G., Pati S.K. (2011). Density functional theoretical investigation of the aromatic nature of BN substituted benzene and four ring polyaromatic hydrocarbons. Phys. Chem. Chem. Phys..

[27] Iijima S. (1991). Helical microtubules of graphitic carbon. Nature.

[28] Dai H. (2002). Carbon Nanotubes: Synthesis, Integration, and Properties. Acc. Chem. Res..

[29] Maniecki T., Shtyka O., Mierczynski P., Ciesielski R., Czylkowska A., Leyko J., Mitukiewicz G., Dubkov S., Gromov D. (2018). Carbon nanotubes: Properties, synthesis, and application. Fibre Chem..

[30] Popov V.N. (2004). Carbon nanotubes: Properties and application. Mater. Sci. Eng..

[31] Seidel R., Graham A.P., Unger E., Duesberg G.S., Liebau M., Steinhoegl W., Kreupl F., Hoenlein W. (2004). High-Current Nanotube Transistors. Nano Lett..

[32] Khazaei A., Soltani Rad M.N., Kiani Borazjani M. (2010). Organic functionalization of single-walled carbon nanotubes (SWCNTs) with some chemotherapeutic agents as a potential method for drug delivery. Int. J. Nanomed..

[33] Hirscher M., Becher M., Haluska M., Quintel A., Skakalova V., Choi Y.-M., Dettlaff-Weglikowska U., Roth S., Stepanek I., Bernier P. (2002). Hydrogen storage in carbon nanostructures. J. Alloys Compd..

[34] Chaban V.V., Prezhdo V.V., Oleg Prezhdo V. (2013). Covalent Linking Greatly Enhances Photoinduced Electron Transfer in Fullerene-Quantum Dot Nanocomposites: Time-Domain Ab Initio Study. J. Phys. Chem. Lett..

[35] Chaban V.V., Pal S., Prezhdo O.V. (2016). Laser-Induced Explosion of Nitrated Carbon Nanotubes: Non-Adiabatic and Reactive Molecular Dynamics Simulations. J. Am. Chem. Soc..

[36] Deb J., Paul D., Sarkar U. (2020). Density Functional Theory Investigation of Nonlinear Optical Properties of T-Graphene Quantum Dots. J. Phys. Chem. A.

[37] Ayoubikaskooli A., Ghaedi A.M., Shamlouei H.R., Saghapour Y. (2022). Influence of donor–acceptor groups on the electrical and optical properties of C50 fullerene. J. Mol. Model..

[38] Carlotti B., Benassi E., Barone V., Consiglio G., Elisei F., Mazzoli A., Spalletti A. (2015). Effect of the π Bridge and Acceptor on Intramolecular Charge Transfer in Push–Pull Cationic Chromophores: An Ultrafast Spectroscopic and TD-DFT Computational Study. Chem. Phys. Chem..

[39] Yong Lee J., Kim K.S., Mhin B.J. (2001). Intramolecular charge transfer of π-conjugated push–pull systems in terms of polarizability and electronegativity. J. Chem. Phys..

[40] Plaquet A., Champagne B., Kulhanek J., Bures F., Bogdan E., Castet F., Ducasse L., Rodriguez V. (2011). Effects of the nature and length of the π-conjugated bridge on the second-order nonlinear optical responses of push–pull molecules including 4,5 dicyanoimidazole and their protonated forms. Chem. Phys. Chem..

[41] Makwani D., Vijaya R. (2007). Frequency-dependent hyperpolarizability of benzene derivatives: Ab-initio calculations. J. Nonlinear Opt. Phys. Mater..

[42] Albert I.D.L., Marks T.J., Ratner M.A. (1997). Large Molecular Hyperpolarizabilities. Quantitative Analysis of Aromaticity and Auxiliary Donor-Acceptor Effects. J. Am. Chem. Soc..

[43] Khan M.S.A. (2016). Srivastava. NH3 and NO2 adsorption analysis of GaN nanotube: A First principle Investigation. J. Electroanal. Chem..

[44] Naderi S., Morsali A., Bozorgmehr M.R., Beyramabadi S.A. (2017). Mechanistic energetic and structural studies of carbon nanotubes functionalised with dihydroartemisinin drug in gas and solution phases. Phys. Chem. Liq..

[45] Smith D.G.A., Burns L.A., Patkowski K., Sherrill C.D. (2016). Revised Damping Parameters for the D3 Dispersion Correction to Density Functional Theory. J. Phys. Chem. Lett..

[46] Silva D.A., Xavier M.J., Dutra J.D.L., Gimenez I.D.F., Freire R.O., da Costa N.B. (2020). Prediction of correct intermolecular interactions in host-guest systems involving cyc Lodextrins. J. Mol. Struct..

[47] Dennington R., Keith T., Millam J. (2009). Gaussview 05.

[48] Frisch M.J., Trucks G.W., Schlegel H.B., Scuseria G.E., Robb M.A., Cheeseman J.R., Scalmani G., Barone V., Petersson G.A., Nakatsuji H. (2009). Gaussian 09.

[49] Fulem M., Laštovka V., Straka M., Ružiĉka K., Shaw J.M. (2008). Heat Capacities of Tetracene and Pentacene. J. Chem. Eng. Data.

[50] Khudhaira A.M., Ajeela F.N., Mohammed M.H. (2019). Theoretical (DFT and TDDFT) insights into the effect of polycyclic aromatic hydrocarbons on Monascus pigments and its implication as a photosensitizer for dye-sensitized solar cells. Microelectron. Eng..

[51] Betowski L.D., Enlow M., Riddick L., Aue D.H. (2006). Calculation of electron affinities of polycyclic aromatic hydrocarbons and solvation energies of their radical anion. J. Phys. Chem. A.

[52] Zouaoui-Rabah M., Sekkal-Rahal M., Djilani-Kobibi F., Elhorri A.M., Springborg M. (2016). Performance of hybrid dft compared to mp2 methods in calculating nonlinear optical properties of divinylpyrene derivative molecules. J. Phys. Chem. A.

[53] Kubas A., Gajdos F., Heck A., Oberhofer H., Elstner M., Blumberger J. (2015). Electronic couplings for molecular charge transfer: Benchmarking CDFT, FODFT and FODFTB against high-level ab initio calculations. Phys. Chem. Chem. Phys..

[54] Ari H., Büyükmumcu Z. (2017). Comparison of DFT functionals for prediction of band gap of conjugated polymers and effect of HF exchange term percentage and basis set on the performance. Comput. Mater. Sci..

[55] Cohen A.J., Handy N.C. (2000). Assessment of exchange correlation functional. Chem. Phys. Lett..

[56] Peach M.J.G., Tellgren E.I., Sałek P., Helgaker T., Tozer D.J. (2007). structural and electronic properties of polyacetylene and polyyne from hybrid and coulomb-attenuated density functionals. J. Phys. Chem. A.

[57] McKechnie S., Booth G.H., Cohen A.J., Cole J.M. (2015). On the accuracy of density functional theory and wave function methods for calculating vertical ionization energies. J. Chem. Phys..

[58] Mardirossian N., Head-Gordon M. (2017). Thirty years of density functional theory in computational chemistry: An overview and extensive assessment of 200 density functionals. Mol. Phys..

[59] Daniel Boese A., Martin J.M.L. (2004). Development of density functionals for thermochemical kinetics. J. Chem. Phys..

[60] Marcano E., Squitieri E., Murgich J., Soscún H. (2012). Theoretical investigation of the static (dynamic) polarizability and second hyperpolarizability of DAAD quadrupolar push–pull molecules. A comparison among HF (TD-HF), DFT (TD-B3LYP), and MP2 (TD-MP2) methods. Comput. Theor. Chem..

[61] Karamanis P., Maroulis G. (2003). Static electric dipole polarizability and hyperpolarizability of fluorodiacetylene. J. Mol. Struct..

[62] Capobianco A., Centore R., Noce C., Peluso A. (2013). Molecular hyperpolarizabilities of push–pull chromophores: A comparison between theoretical and experimental results. Chem. Phys..

[63] Fonseca T.L., de Oliveira H.C.B., Amaral O.A.V., Castro M.A. (2005). MP2 static first hyperpolarizability of azo-enaminone isomers. Chem. Phys. Lett..

[64] Champagne B., Perpète E.A., Jacquemin D. (2000). Assessment of Conventional Density Functional Schemes for Computing the Dipole Moment and (Hyper)polarizabilities of Push-Pull π-Conjugated Systems. J. Phys. Chem. A.

[65] Günay N., Pir H., Avcı D., Atalay Y. (2013). NLO and NBO Analysis of Sarcosine-Maleic Acid by Using HF and B3LYP Calculations. J. Chem..

[66] Heesink G.J.T., Ruiter A.G.T., van Hulst N.F., Bolger B. (1993). Determination of Hyperpolarizability Tensor Components by Depolarized Hyper Rayleigh Scattering. Phys. Rev. Lett..

[67] Labidi N.S. (2016). Semi empirical and Ab initio methods for calculation of polarizability (α) and the hyperpolarizability (β) of substituted polyacetylene chain. Arab. J. Chem..

[68] Limacher P.A., Mikkelsen K.V., Lüthi H.P. (2009). On the accurate calculation of polarizabilities and second hyperpolarizabilities of polyacetylene oligomer chains using the CAMB3LYP density functional. J. Chem. Phys..

[69] Yanai T., Tew D.P., Handy N.C. (2004). A new hybrid exchange–correlation functional using the Coulomb-attenuating method (CAM-B3LYP). Chem. Phys. Lett..

[70] Ma N.N., Yang G.C., Sun S.L., Liu C.G., Qiu Y.Q. (2011). Computational study on second-order nonlinear optical (NLO) properties of a novel class of two-dimensional Λ- and W-shaped sandwich metallocarborane-containing chromophores. J. Organomet. Chem..

[71] Kirtman B., Champagne B., Bishop D.M. (2000). Electric Field Simulation of Substituents in Donor-Acceptor Polyenes: A Comparison with Ab Initio Predictions for Dipole Moments, Polarizabilities, and Hyperpolarizabilities. J. Am. Chem. Soc..

[72] Winkler R., Pantelides S.T. (1997). Charge transfer and dipole moments of polyatomic systems. J. Chem. Phys..

[73] Del Zoppo M., Castiglioni C., Gerola V., Zuliani P., Zerbi G. (1998). Effect of bond length alternation and of bond length alternation oscillations on the molecular nonlinear optical response of push–pull polyenes. J. Opt. Soc. Am..

[74] Benchehaima F.Z., Springborg M., Sekkal Rahal M. (2022). Nonlinear optical properties DFT calculations of polyacethylene and copolymers models substituted with aldimines chromophores as side chains. J. Comput. Chem..

[75] Thanthiriwatte K.S., de Silva K.M.N. (2002). Non-linear optical properties of novel fluorenyl derivatives—Ab initio quantum chemical calculations. J. Mol. Struct..

